# Global Transcriptional and Phenotypic Analyses of *Escherichia coli* O157:H7 Strain Xuzhou21 and Its pO157_Sal Cured Mutant

**DOI:** 10.1371/journal.pone.0065466

**Published:** 2013-05-30

**Authors:** Hongqing Zhao, Chen Chen, Yanwen Xiong, Xuefang Xu, Ruiting Lan, Haiyin Wang, Xinyue Yao, Xiangning Bai, Xuetong Liu, Qiong Meng, Xiaoai Zhang, Hui Sun, Ailan Zhao, Xuemei Bai, Yuli Cheng, Qiang Chen, Changyun Ye, Jianguo Xu

**Affiliations:** 1 State Key Laboratory for Infectious Disease Prevention and Control, Collaborative Innovation Center for Diagnosis and Treatment of Infectious Disease, National Institute for Communicable Disease Control and Prevention, Changping, Beijing, China; 2 School of Biotechnology and Biomolecular Sciences, University of New South Wales, Sydney, New South Wales, Australia; 3 Beijing Center for Disease Prevention and Control, Beijing, China; U. S. Salinity Lab, United States of America

## Abstract

*Escherichia coli* O157:H7 is an important food-borne pathogen that can cause hemorrhagic colitis and hemolytic-uremic syndrome in humans. pO157_Sal, a novel conjugative plasmid is present in a Chinese O157:H7 outbreak strain Xuzhou21. Here we investigated the phenotypic and transcriptional differences between the wild type strain Xuzhou21 and the pO157_Sal cured mutant strain Xuzhou21m. RNA-Seq analysis found that all 52 ORFs encoded on pO157_Sal were transcribed. One hundred and sixty eight chromosomal and pO157 genes were differentially expressed (≥2 fold difference) between Xuzhou21 and Xuzhou21m. Sixty-seven and 101 genes were up-regulated and down-regulated respectively by pO157_Sal including genes related to stress response, adaption and virulence. The plasmid-cured mutant Xuzhou21m grew slower than wild type Xuzhou21 and pO157_Sal plasmid complemented strain Xuzhou21c in M9 medium under the condition of high NaCl or presence of sodium deoxycholate (NaDC), corroborating with the RNA-Seq data. Seven differentially expressed genes are associated with NaDC resistance, including the adenine-specific DNA-methyltransferase gene (*dam*), multidrug efflux system subunit gene *mdtA*, hyperosmotically inducible periplasmic protein gene *osmY* and oxidation-reduction related genes while two differentially expressed genes (*osmY* and *pspD*) are likely to be related to resistance to osmotic pressure. A number of differentially expressed genes were virulence associated including four genes encoding T3SS effectors from the chromosome and *ehxD* from pO157. Through complementation of Xuzhou21m with a plasmid construct carrying the pO157_Sal *hha* homolog we further showed that the pO157_Sal *hha* represses the expression of T3SS effectors. These findings demonstrated that the plasmid pO157_Sal affects the transcription of the chromosomal and pO157 plasmid genes and contributes to the enhanced ability to resist stress. We conclude that pO157_Sal plays an important role in regulating global gene expression and affects the virulence and adaptation of *E. coli* O157:H7.

## Introduction

The enterohemorrhagic *Escherichia coli* (EHEC) O157:H7 causes acute gastroenteritis, hemorrhagic colitis and hemolytic-uremic syndrome (HUS) in humans [1], [2]. The latter is a severe infection sequelae characterized by thrombotic microangiopathy, hemolytic anaemia and acute renal failure which can lead to long-term kidney damage or fatal outcome [1], [2]. *E.coli* O157:H7 was first recognized as an important human pathogen in 1982 during an investigation of a food-borne disease outbreak in the United States [3] and has caused many outbreaks in the past three decades, with a wide range of clinical illness [4]. In 2006, an outbreak associated with spinach caused high rates of bloody diarrhea (22/23), hospitalization (13/23) and HUS (7/23) [5], suggesting that the outbreak strain, TW14359, has evolved to higher virulence [6]. Further studies have shown that TW14359 expresses higher levels of many virulence genes and a range of other chromosomal and pO157-encoded genes. It has also been shown that TW14359 has better adherence to epithelial cells than *E.coli* O157:H7 strain Sakai [7], [8].

An outbreak of *E. coli* O157:H7 occurred in Xuzhou, China in 1999, causing about 20,326 infections, 195 hospitalized HUS patients and 177 deaths [9]. Our group has recently sequenced the outbreak strain Xuzhou21 [10]. We also discovered that a novel conjugative plasmid pO157_Sal was present in Xuzhou21 and was found nearly unique in the outbreak isolates in China [11]. The pO157_Sal contains 52 ORFs and has a full set of genes for the type IV secretion system (T4SS), but no known virulence-related genes were identified. Several genes found on pO157_Sal are homologous to transcriptional regulatory genes such as *stpA* and *hha* ¸ both of which have been reported to be implicated in virulence or environmental adaptation [12], [13], [14], [15]. In addition, the pO157_Sal *mpr* gene is homologous to *stcE* on pO157 which encodes a zinc metalloproteinase and may play a role in adherence [12]. We further showed that Xuzhou21 has the capacity to provoke elevated proinflammatory responses with levels of IL-6 and IL-8 induction being significantly higher than that induced by EDL933. Xuzhou21 also carries a highly inducible Stx2 prophage [16].

RNA-Seq (whole transcriptome shotgun sequencing) is a next generation sequencing platform based assay of genome-wide bacterial gene expression [17]. RNA-Seq can reveal the entire transcriptional landscape and has less systematic bias compared to microarray technology [18]. To further enhance our understanding of the Xuzhou outbreak strain and to investigate the global effects of pO157_Sal on the gene expression, virulence and adaptation of Xuzhou21, we cured the pO157_Sal plasmid from Xuzhou21 and compared the transcriptomic differences by RNA-Seq between the wild-type strain Xuzhou21 and the pO157_Sal cured strain Xuzhou21m.

## Results

### Plasmid curing and complementation

**Figure 1.Transcriptomic:**
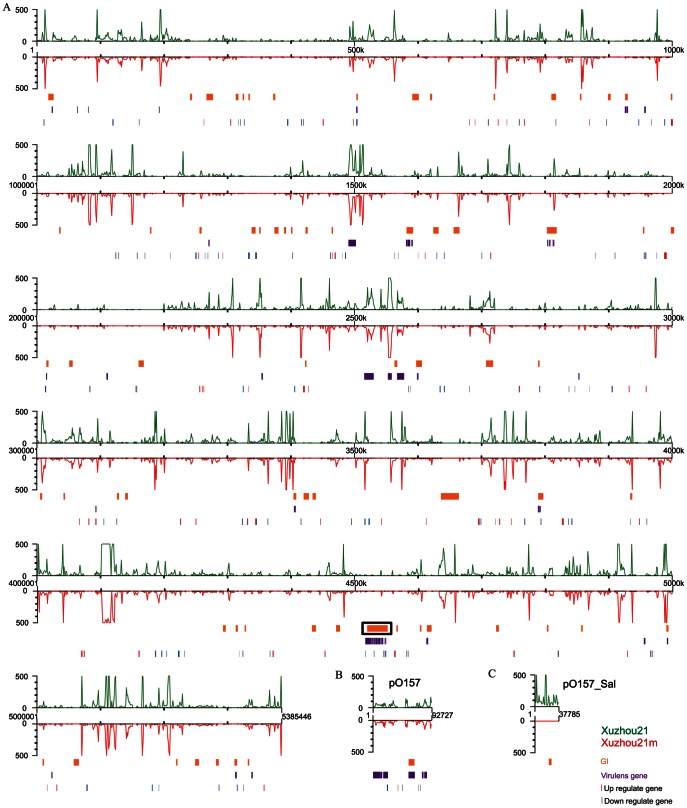
Transcriptomic maps of Xuzhou21 and pO157_Sal cured Xuzhou21m. A-C. Transcriptomic comparisons of the chromosome, pO157 plasmid and pO157_Sal plasmid between Xuzhou21 and Xuzhou21m respectively. The number of reads covering a position was plotted along the genome (X-axis). Xuzhou21 and Xuzhou21m are indicated in green and red respectively. Fold of coverage is shown at a maximum of 500 (Y-axis). Genomic islands (GIs) and major virulence genes are indicated in orange and purple with the LEE locus marked with a rectangular box. Up-regulated and down-regulated genes are indicated in red and blue vertical bar below the X-axis respectively.

Xuzhou21 was treated with SDS and high temperature to cure the pO157_Sal plasmid and curing was confirmed by PCR using primer pairs, ehxA-F/ehxA-R and p247-F/p247-R, targeting *ehxA* and *traL* specific for amplification of pO157 and pO157_Sal respectively. Xuzhou21 was positive for these two genes while the pO157_Sal cured strain Xuzhou21m was only positive for *ehxA*. We found that the efficiency of curing pO157_Sal plasmid was 1%∼3%. We further sequenced Xuzhou21m using Illumina sequencing to confirm that no other changes occurred in the genome during plasmid curing. Reads were mapped to Xuzhou21 genome with an average 114-fold coverage. No insertion or deletion was found and plasmid pO157 was intact in Xuzhou21m. No DNA fragment from plasmid pO157_Sal was detected in Xuzhou21m **(** **Figure 1** **)**. Fifty-eight tentative SNPs were identified. However, using RNA-Seq data, only two non-synonymous SNPs, one each from two genes of unknown function (CDCO157_2530 [C to A] and CDCO157_2534 [G to T]), were confirmed to be genuine.

We further created a pO157_Sal complemented strain, Xuzhou21c. The plasmid pO157_Sal was first marked with the kanamycin resistance gene using one-step gene inactivation and was transformed into Xuzhou21m successfully. The presence of the pO157_Sal in this complemented strain Xuzhou21c was confirmed by PCR using pO157_Sal specific primer pair, p247-F and p247-R.

Q-PCR was used to determine the relative copy number of pO157 and pO157_Sal in Xuzhou21, Xuzhou21m using 2^−ΔΔCT^ method [19]. The target genes on pO157 and pO157_Sal were *espP* and *traL*. The reference chromosomal gene was g*apA*. Q-PCR showed that the two strains had 2.35±0.49 and 2.21±0.24 copies of pO157 respectively. The copy number of pO157_Sal in Xuzhou21 was 2.04±0.18. These results suggested that the two plasmids have similar copy numbers and the curing of pO157_Sal has no effect on the copy number of pO157.

### Transcriptional profiling of Xuzhou21 and Xuzhou21m

To determine the effect of pO157_Sal at transcriptomic level, the transcriptomes of Xuzhou21 and Xuzhou21m were compared using RNA-Seq. Cells were cultured in LB broth to exponential phase and mRNA were sequenced using Illumina sequencing **(** **Figure 1** **)**. About 6.5 million reads were obtained for both Xuzhou21 and Xuzhou21m, giving a 212 fold coverage respectively. About 1.9 and 1.4 million “clean reads” were obtained for Xuzhou21 and Xuzhou21m respectively after filtering out any reads with more than 10% uncalled bases (Ns) or with more than 50% of bases with a base quality less than 5.0. The reads were mapped to Xuzhou21 reference genome. Allowing for up to 2 mismatches for a read, 67.9% of Xuzhou21 and 63.35% Xuzhou21m reads were mapped to the Xuzhou21 genome (**Table 1**). The reads covered 70.13% and 65.6% gene regions for Xuzhou21 and Xuzhou21m respectively. Sequence reads located in intergenic regions account for 28.28% and 29.89% of all sequence reads obtained for Xuzhou21 and Xuzhou21m respectively. Although expression of intergenic regions may have important information, these reads were not analyzed further.

**Table 1:** Mapping statistics of RNA-Seq reads to the strains.

	Xuzhou21*		Xuzhou21m*	
	No. of reads	Total length (bp)	No. of reads	Total length (bp)
**Total reads obtained**	6,499,414		6,488,441	
**Reads mapped to the reference genome**	1,901,206	171,108,540	1,436,791	129,311,190
**Reads mapped exactly**	1,667,789	150,101,010	1,256,612	113,095,080
**Reads mapped with 1 mismatch**	196,774	17,709,660	150,944	13,584,960
**Reads mapped with 2 mismatch**	36,643	3,297,870	29,235	2,631,150
**Reads mapped to genes**	1,177,730	105,995,700	873,828	78,644,520
**Reads mapped to integenic regions**	537,625	424,768	429,402	388,103

* Xuzhou21 was used as reference with a size of 5,515,958 bp including 4,735,414 bp coding and 780,544 bp intergenic regions.

As a result, a high-resolution transcriptomic map of the Xuzhou21 genome was generated. The level of gene expression was calculated using reads per kilobase per million reads (RPKM) method [20]. Since the reproducibility was relatively low in genes with low RPKM value [21], only genes with RPKM≥10 were considered expressed. We found that 3991 of 5214 genes from Xuzhou21 had a RPKM greater than ten, 963 genes have a RPKM greater than zero but less than ten, and 260 genes had no reads (RPKM = 0). Ninety-five percent of the genes of Xuzhou21 genome had an RPKM>0 and were thus transcribed. One hundred and sixteen genes (2.22%) had a RPKM greater than 1,000 representing highly expressed protein coding genes, among which, 42 and 24 encode ribosomal proteins and hypothetical proteins respectively. Other highly transcribed genes including genes encoding elongation factor Tu, MokW, cold shock protein CspE and flagellin. MokW is only 51 amino acids long and little is known of its function. Of the 1223 genes with RPKM less than ten, 432 (35.32%) were genes of unknown function and a further 369 (30.17%) had putative functions.

**Figure 2:**
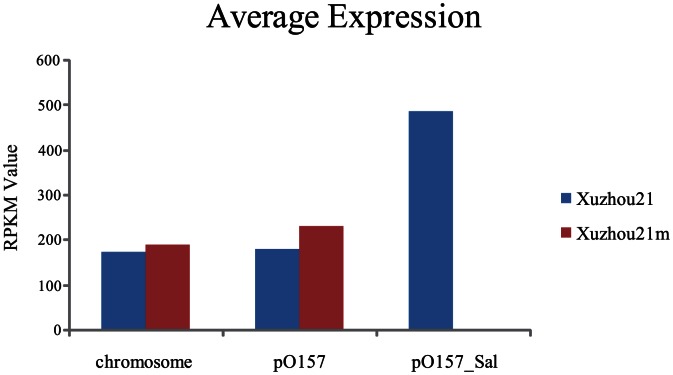
Transcriptional levels of Xuzhou21 and pO157_Sal cured Xuzhou21m. Average level of gene expression (RPKM value) of the two strains by chromosome, pO157 and pO157_Sal as indicated.

Of the 5070 chromosomal genes and 92 pO157 plasmid genes, 4814 (94.95%) and 88 (95.65%) were transcribed respectively while all pO157_Sal genes were transcribed in Xuzhou21. In contrast, 94.62% (4749/5070) and 95.65% (88/92) of the chromosomal and pO157 genes were expressed In Xuzhou21m respectively. Interestingly, pO157_Sal genes showed much higher level of expression on average than genes on the chromosome and pO157 **(** **Figure 2** **)**.

### Validation of RNA-Seq results using RT-qPCR

**Figure 3.Validation:**
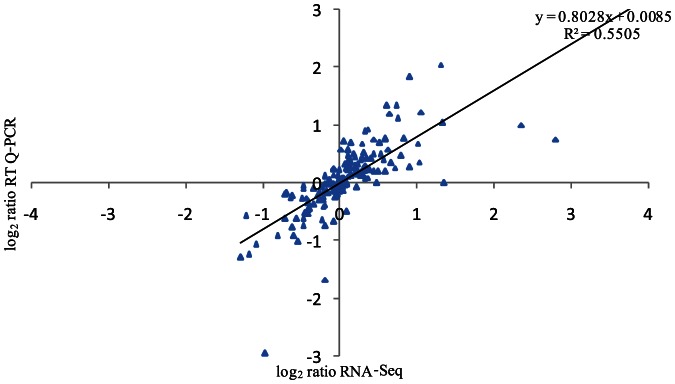
of RNA-Seq results by RT-qPCR. Plot of gene expression (fold change) determined by the RNA-Seq (Y-axis) and RT-qPCR (X-axis) for 167 selected genes (Pearson's correlation, R^2^ = 0.5505, *P*<0.01). The fold change was expressed as log_2_ values.

To validate RNA-Seq results, 167 genes were selected to validate their level of expression using RT-qPCR. We selected 51 highly (RPKM>500), 72 moderately (500>RPKM>100) and 44 poorly (100>RPKM>10) expressed genes to encompass the wide spectrum of variation in expression levels. As shown in **Figure 3**, RT-qPCR data is consistent with the RNA-Seq data (R^2^ = 0.5505, *P*<0.01). The slope of the trend line as shown in **Figure 3** is less than 45 degrees, indicating that RNA-Seq is generally less sensitive than RT-qPCR for quantification of gene expression. Thus RNA-Seq gave a conservative estimate of the number of differentially expressed genes.

### Differentially expressed genes between Xuzhou21 and Xuzhou21m

There were 3733 genes with RPKM ≥10 in both strains as candidates for differential expression analysis. To provide a reliable comparison, genes with RPKM less than10 in both strains were excluded. However genes that had an RPKM<10 in one strain but were highly expressed (RPKM ≥10) in the other (231 in Xuzhou21 and 265 in Xuzhou21m) were included as candidates [22]. Consequently, 168 genes with 2-fold change or greater and *P*<0.05 were identified as significantly differentially expressed genes. These genes are listed in **Table S1** along with their functions and their levels of expression. Excluding the 52 genes located on pO157_Sal, 67 genes were up-regulated and 101 genes were down-regulated in Xuzhou21. In the 67 up-regulated genes, the expression level of 24 genes in Xuzhou21m was zero; and in the 101 down-regulated genes, the expression level of 12 genes in Xuzhou21 was zero. The vast majority of the differentially expressed genes were chromosomal. Five genes were from pO157 including three down-regulated and two up-regulated genes. Sixteen of the up-regulated genes were hypothetical proteins with unknown functions, while 28 of the down-regulated genes were hypothetical proteins.

The up-regulated genes in Xuzhou21 were in 15 gene function classes based on cluster of orthologous genes (COG) classification **(Table S1)**. Eleven up-regulated genes belong to the COG classes of transport and metabolism of amino acids, carbohydrates, inorganic ions, lipids and nucleotides and six in the class of energy production and conversion. Two genes encoding pilus/fimbrial assembly proteins, FimA and PilN, and a gene involved in curli production (CsgG) were up-regulated in Xuzhou21. Curli is associated with biofilm formation, adhesion and invasion [23], [24]. It is interesting to note that several genes are related to stress response including heat shock chaperone gene *ibpB*, cold shock protein gene *cspE*, hyperosmotically inducible periplasmic protein gene *osmY*, peripheral inner membrane phage-shock protein gene *pspD*, multidrug efflux system subunit gene *mdtA*, adenine-specific DNA-methyltransferase gene *dam* and oxidation-reduction related genes, suggesting that pO157_Sal enhances expression of these genes which play a role in stress response.

The down-regulated genes also encompass a range of COG gene function classes. Twenty three down-regulated genes belong to the COG classes of transport and metabolism of amino acids, carbohydrates, inorganic ions, lipids and nucleotides. Five genes are related to virulence including *ler*, *ehxD*, 3 LEE effector genes and 1 non-LEE effector gene. Five genes were in the class of cell motility and are involved in pilus biogenesis and assembly, which may also affect virulence. Thus, the pO157_Sal has a repression effect on the expression of these metabolic and virulence associated genes.

### pO157_Sal enhances hyperosmosis and bile salt resistance

**Figure 4:**
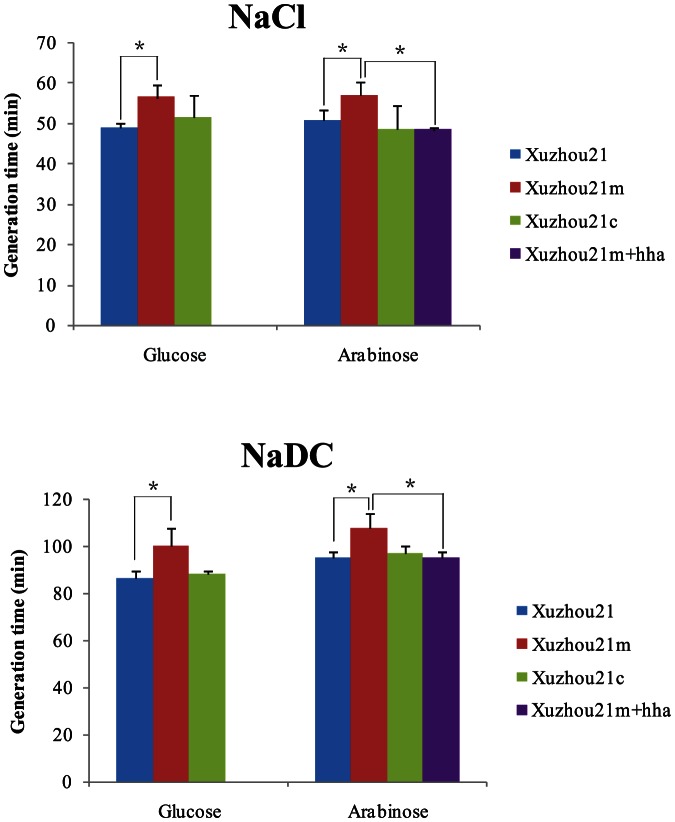
Comparison of generation time of the mutants with the wild type under different conditions. Xuzhou21, Xuzhou21m, Xuzhou21c and Xuzhou21+*hha* are indicated in blue, red, green and violet respectively. The generation time in M9 basal medium containing 0.2% (w/v) glucose as carbon source or 0.2% (w/v) arabinose as carbon source and *hha* expression inducer, and 0.37% NaCl or 0.67% (w/v) NaDC respectively. Error bars represent the standard errors from three separate growth experiments. Statistical significance (p<0.05) between Xuzhou21m and other strains is indicated by an asterisk (*).

Since the RNA-Seq data showed that several genes related to stress responses were more highly expressed in Xuzhou21, which suggests that pO157_Sal plays a role in stress response, we tested resistance to bile salt and osmotic pressure. The growth rates of Xuzhou21, Xuzhou21m and Xuzhou21c in the M9 basal medium with 0.2% glucose were the same. In contrast, the growth rates of these three strains in the M9 medium containing an additional 0.37% NaCl or 0.67% NaDC were different. The generation time of Xuzhou21 and Xuzhou21m when cultured in M9 containing the additional NaCl, were 48.8±1.25 minutes and 56.3±3.21 minutes (*t* test, *P* = 0.048) respectively, while the generation time of Xuzhou21 and Xuzhou21m when cultured in M9 containing NaDC were 86.3±3.40 minutes and 100.0±7.50 minutes respectively (*t* test, *P* = 0.016) respectively. The generation times of Xuzhou21c when cultured in M9 containing NaCl and NaDC were 51.3±5.51 minutes and 88.3±1.44 minutes respectively and were similar to those of Xuzhou21 **(** **Figure 4** **)**. Therefore, pO157_Sal promoted the growth of Xuzhou21 significantly under both conditions.

### The effect of pO157_Sal encoded global gene regulator Hha

Analysis of the genes on pO157_Sal found that only two known homologs of global gene regulators *stpA* and *hha* that may play the role in hyperosmosis and bile resistance in Xuzhou21. We attempted to test this by constructing a plasmid carrying a pO157_Sal *stpA* or *hha*. We initially used a constitute expression vector pMD®20 T (TAKARA). However, the empty T vector has a high background effect on Xuzhou21m. We then used an arabinose inducible vector pBAD30, which was found to has no effect on Xuzhou21m, to create pBAD30 constructs carrying the pO157_Sal *stpA* and *hha* separately and transformed to Xuzhou21m as Xuzhou21m+*stpA* and Xuzhou21m+*hha* respectively. However, Xuzhou21m+*stpA* did not grow under arabinose induction. We then tested resistance to osmotic pressure and bile salt of Xuzhou21m+*hha*. As shown in **Figure 4**, Xuzhou21, Xuzhou21c and Xuzhou21+*hha* grew similarly while Xuzhou21m grew significantly slower than the other three strains (t test, *P*<0.05).

We further tested whether *hha* affects the expression of the LEE associated T3SS genes, six of which *ler*, *ehxD*, 3 LEE effector genes (*espH, espF, espG*) and 1 non-LEE effector gene (*espY3*) were differentially expressed based on RNA-Seq data. The expression of *espF* and *espH* in Xuzhou21m+*hha* grown in LB to late exponential phase (OD_600_ = 3.0) and under induction of 0.05% arabinose were measured by qPCR. The transcription levels of *espF* and *espH* in Xuzhou21m were 3.2±0.44 (*t* test, *P* = 0.001) and 1.9±0.27 times (*t* test, *P* = 0.003) higher than Xuzhou21. The transcription levels of *espF* and *espH* in Xuzhou21m+*hha* were reduced to 0.5±0.06 (*t* test, *P* = 0.003) and 0.3±0.04 times (*t* test, *P* = 0.001) of those of Xuzhou21.

## Discussion

To understand further the role of the novel plasmid pO157_Sal, we created a pO157_Sal cured Xuzhou21m strain for comparative studies. Using RNA-Seq, a high-resolution transcriptomic map of Xuzhou21 was constructed which showed that 95% of the genes were transcribed (RPKM>0). The expression of over 168 genes including 163 genes from the chromosome and 5 genes from the pO157 plasmid were affected by plasmid pO157_Sal. Thus the presence of pO157_Sal has a far reaching effect on the biology of Xuzhou21. pO157_Sal specific genes in Xuzhou21 were all transcribed. Notably, pO157_Sal genes showed much higher expression level on average than other genes in the chromosome and pO157.

pO157_Sal affects bile salt resistance since Xuzhou21m grew 16% slower than Xuzhou21 in the presence of bile salt. The growth rate of the complemented strain Xuzhou21c was restored to the same level as the wild type. Seven genes/systems that were up-regulated by pO157_Sal can be associated with this role including multidrug efflux system subunit gene *mdtA*, the adenine-specific DNA-methyltransferase gene *dam*, hyperosmotically inducible periplasmic protein gene *osmY* and oxidation-reduction related genes **(** **Table 2** **)**. The multidrug efflux system encoded by *mdtABCD* locus could expel bile salt from the cytoplasm after they breach the cell membrane and thus increase resistance to bile salt [25]. The adenine-specific DNA-methyltransferase coded by *dam* is important for bile resistance by controlling integrity of the envelope as shown in *Salmonella enterica* [26]. *dam* mutants are sensitive to bile salt mostly in exponential-growth phase [27]. Four genes related to oxidation-reduction may also affect bile salt resistance since proteins involved in oxidation-reduction reactions are differentially expressed after exposure to bile salt [28].

**Table 2:** The differential expressed genes related to resistance to bile salt or NaCl stress.

Gene ID	Name	Fold change		Function	reference
		Log_2_ RNA-Seq	Log_2_ Q-PCR		
**bile salt resistance related genes**					
CDCO157_2659	*mdtA*	1.07	1.78	multidrug efflux system subunit	[25]
CDCO157_3874	*dam*	1.62	1.52	Putative adenine-specific DNA-methyltransferase	[27]
CDCO157_5020	*osmY*	1.21	1.58	hyperosmotically inducible periplasmic protein	[29]
CDCO157_2169	*rnfA*	2.07	2.51	Predicted NADH:ubiquinoneoxidoreductase, subunit	[28]
CDCO157_2173	*rnfG*	1.32	1.24	Predicted NADH:ubiquinoneoxidoreductase, subunit	
CDCO157_3512	oxidoreductase	1.14	1.04	NADPH-dependent glutamate synthase beta chain and related oxidoreductases	
CDCO157_3643	oxidoreductase	1.23	1.67	Fe-S oxidoreductase	
**Hyperosmosis resistance related genes**					
CDCO157_5020	*osmY*	1.21	1.58	hyperosmotically inducible periplasmic protein	[62]
CDCO157_1804	*pspD*	∞	8.99	peripheral inner membrane phage-shock protein	[30], [63]

pO157_Sal also affects resistance to osmotic pressure as shown by growth rate difference. Differentially expression of two genes (*osmY* and *pspD*) related to osmotic resistance was observed. *osmY* encodes a periplasmic protein OsmY which is commonly involved in osmotic stress and resistance to bile salt [29]. *pspD* and the other 4 genes in the *psp* operon was up-regulated by pO157_Sal although only *pspD* reached the cutoff. The major function of the *psp* regulon is to stabilize and maintain proton motive force within a stressed cell [30] and thus may also promote resistance to osmotic pressure.

In addition, heat shock chaperone gene *lpbB* and cold shock protein gene *cspE* related to heat and cold resistance respectively were up-regulated in Xuzhou21. *E. coli* cold shock protein CspA family consists of nine proteins (CspA to CspI). CspE and CspC are constitutively produced at 37°C and up-regulate the expression of the gene encoding global stress response regulator RpoS through *rpoS* message stabilization [31]. In addition, CspE functions as a negative regulator for *cspA* [32]. CspA alters the secondary structure of RNA, making it more susceptible to degradation [33].

Very few virulence-associated genes were found to be differentially expressed between Xuzhou21 and Xuzhou21m **(Table S3)**, which may be related to the exponential growth phase and the medium we used. Previous studies show that LEE1-3 genes usually express much higher than exponential phase in the transition from the late exponential phase to the stationary phase [34], [35]. In the stationary phase, the expression of LEE-encoded genes are down-regulated [36]. For examples, the *ler* promoter activity increased greatly from the mid-exponential phase to the stationary phase. Ler directly regulates genes within the LEE PAI as well as genes elsewhere in the genome [37]. Therefore, genes located on LEE were up-regulated in late exponential phase via *ler*. Interestingly pO157_Sal represses *ler* and four LEE and non-LEE encoded effectors as described above, consistent with repression of virulence genes during late exponential growth reported previously [34], [35]. It would be interesting to examine the effect of pO157_Sal on these genes in the stationary phase. Previous studies have shown that virulence genes are up-regulated when O157:H7 is grown in minimal medium [38]. The expression of virulence genes of O157:H7 have also used DMEM [39] and LB medium [40] previously. Future studies will be conducted using other media to compare with expression in LB to shed further light on the effects of pO157_Sal on the expression of virulence genes.

Additionally, there are 7 genes related to bacteria motility including 5 down-regulated (*hofC, yadN, papD, fimL* and major tail protein V gene) and 2 up-regulated (*pilN* and *fimA*) genes by pO157_Sal. But no difference in motility between Xuzhou21 and Xuzhou21m was found when conventional soft agar and U-shape tube motility assay was performed (data not shown).

pO157_Sal clearly has a major effect on the expression of chromosomal and pO157 genes, in particular genes associated with cell metabolism. Since a large number of genes of different functions were differentially expressed and these genes are distributed across the chromosome, the effects of pO157_Sal on these genes must be exerted through regulation by global regulatory genes present on pO157_Sal. There are two genes homologous to global regulatory genes, *stpA* and *hha* on the pO157_Sal plasmid.

Previous studies showed that the chromosomal homolog of *stpA* plays a similar but minor role to H-NS, both of which bind DNA nonspecifically as global gene regulators with certain differential effect [41], [42]. The differential effect may be associated with their intracellular concentration of which H-NS is higher than StpA [43]. There are 2 *stpA* homologs on the chromosome (CDCO157_3291 and CDCO157_1668). Both homologs had no transcriptional difference between Xuzhou21 and Xuzhou21m with RPKM values of 1035.06 and 898.659 for CDCO157_1668 and 283.562 and 275.738 for CDCO157_3291 respectively. The pO157_Sal plasmid copy of *stpA* is homologous to the two chromosomal copies (the pO157_Sal *stpA* shares 46% and 43% DNA identity with CDCO157_3291 and CDCO157_1668 respectively). The RPKM value of pO157_Sal *stpA* is much higher (2818.8) and may play a functionally similar but larger role. Studies have also shown that the expression of the chromosomal *stpA* in *E. coli* K-12 is up-regulated by high osmolarity during cell growth [44], further pointing to a regulatory role of StpA in the resistance to osmotic pressure. Unfortunately, Xuzhou21m+*stpA* did not grow in M9 when *stpA* expression was induced using arabinose and thus the function role of *stpA* could not be confirmed. The failure to grow may be associated with overexpression of *stpA*which can orgnize DNA into compact higher order structure via the magnesium in M9 medium [45].

Hha could form heteromeric complexes with H-NS and StpA [46] and have multiple effects on several virulence or adaptation associated genes [13], [14], [47]. Hha enhances the ability of H-NS to repress the *ehx* operon on the pO157 [48], [49] and mediates repression of *ler* leading to reduced expression of the *esp* operon [13]. Hha also controls biofilm formation by repressing the transcription of rare codon tRNAs [50] and repressing transcription of fimbrial genes [51]. RNA-Seq showed that both *ler* and *ehxD* were repressed by pO157_Sal. The transcription of *espF* and *espH* was repressed by Hha in Xuzhou21m+*hha*. Therefore we conclude that the *hha* located on pO157_Sal represses the transcription of the LEE encoded T3SS and its effectors. Both the whole pO157_Sal plasmid and single gene (*hha*) complementations of Xuzhou21m showed that *hha* plays a role in the resistance to NaCl and bile salt.

It is interesting to note that there is also a *hha* homolog present in pO157 as well as on the chromosome. The pO157 *hha* homolog (pO157_0071) shares 37% amino acid identity with the pO157_Sal *hha*, although the two homologs have no significant similarity at DNA sequence level. The RPKM values of the chromosomal copy, CDCO157_0501, were 845.206 and 1022.92 in Xuzhou21 and Xuzhou21m respectively, indicating no transcriptional difference. In contrast, the RPKM values of the pO157 copy, CDCpO157_0071, were 117.155 and 0 in Xuzhou21 and Xuzhou21m respectively, and it seems that pO157_Sal turns on the expression of pO157_0071. Therefore, even if pO157_0071 plays a similar role to the pO157_Sal *hha* it is conditional upon the activation by pO157_Sal.

Previous study have shown that *hha* regulates the expression of *csgD* which is a positive regulator of curli genes encoded by *csgDEFG* and *csgBAC* operons [52]. However, the RNA-Seq data showed some opposing effects of the pO157_Sal *hha* on the curli genes, *csgG* of the *csgDEFG* operon was up-regulated in Xuzhou21 while *csgA* of the *csgBAC* operon was down-regulated in Xuzhou21. The RPKM values of *csgD* had no difference between Xuzhou21 and Xuzhou21m, suggesting that the Hha regulation is independent of CsgD. Overall the expression of both operons was low with RPKM values on average of 8.42 and 8.87 in Xuzhou21 and Xuzhou21m respectively. This may be due to the growth phase of the cells we used for RNA-Seq as curli fimbriae are usually expressed higher during the stationary phase [53].

The pO157_Sal *mpr* gene encodes a putative zinc metalloproteinase, which is homologous to *stcE* on pO157. StcE plays a role in intimate adherence of *E.coli* O157:H7 to HEp-2 cells [12] by cleavage of glycoproteins from the cell surface. Thus, the Mpr homolog encoded on pO157_Sal may also be involved in adherence. Future studies will assess the role of the *mpr* gene in virulence.

In conclusion, plasmid pO157_Sal affected the expression of 168 genes involved in a range of functions under the conditions tested and may contribute to the resistance to NaCl and NaDC. The wide-ranging effect we observed is likely to be a result of gene regulation by global regulators encoded on the plasmid. The *stpA* and *hha* homologs on pO157_Sal are the most likely regulators playing this role and we have confirmed the effect of *hha* on resistance to NaCl and NaDC. Further studies will be needed to determine the effects by these regulators specifically and their effects on virulence.

## Materials and Methods

### Bacterial strains, plasmids and growth conditions

The *E. coli* O157:H7 strain Xuzhou21 was isolated from feces of a HUS patient from an outbreak in China in 1999 [11].The nonpathogenic *E.coli* K12 MG1655 was used as negative control. The bacteria were routinely grown in Luria-Bertani (LB) broth or on LB agar plates (pH 7.2). Chloramphenicol (35 µg/ml), kanamycin (40 µg/ml), ampicillin (100 µg/ml) and arabinose (0.05%) were added as required.

For growth assays, the bacteria were incubated overnight in 5 ml LB broth at 37°C, and were collected and washed with PBS (137 mM NaCl, 2.7 mM KCl, 10 mM Na_2_HPO_4_ and 2 mM KH_2_PO_4_, pH 7.2–7.4) for three times. The bacteria were adjusted to about OD_600_ = 0.90 with PBS and 40 µl were inoculated into 4 ml M9 minimal medium (12.8 g/L Na_2_HPO_4_, 3.0 g/L KH_2_PO_4_, 0.5 g/L NaCl, 1.0 g/L NH_4_Cl, 2 mM MgSO_4_, 0.1 mM CaCl_2_ and 0.001% thiamine), with the addition of 0.2% glucose or 0.2% arabinose (for gene complemented strains and pBAD30 negative control). Additional differential compositions were NaDC (0.67%) to test bile salt resistance or NaCl (0.37%) to test osmotic stress resistance. We tested a range of NaDC and NaCl concentrations initially. The concentration gave the best difference and thus were selected for the final growth assays. The growth was measured as optical density at 600 nm at hourly intervals. All assays were performed in triplicate, and the results were expressed as mean± standard deviation (SD).

### Curing of plasmid pO157_Sal

The plasmid pO157_Sal was removed from Xuzhou21 using sodium dodecyl sulfate (SDS) [54] and high temperature treatment [55]. Briefly, steps were (i) Xuzhou21 was inoculated in 5 ml LB broth at 37°C with shaking for 16 h. (ii) 50 µl of the above culture was inoculated in 5 ml fresh LB broth with 0.05% SDS at 37°C with shaking for 16 h. (iii) 50 µl of the above culture was inoculated in 5 ml fresh LB broth at 42°C with shaking for 16 h. Repeat steps (ii) and (iii), and then 50 µl of the culture was inoculated in 5 ml fresh LB broth at 37°C with shaking for 16 h. The culture was then 10-fold serial diluted and spread on LB plates in order to obtain single colonies. The PCR primers used to identify the plasmid pO157_Sal were p247-F (5′-AGCGCATCGCTACAAGCACA-3′) and p247-R (5′-ACGACAACCCCACCGAGGCT-3′) [11]. The PCR primers used to identify the pO157 were ehxA-F (5′-AGCTGCAAGTGCGGGTCTG-3′) and ehxA-R (5′-TACGGGTTATGCCTGCAAGTTCAC-3′). The plasmid and chromosomal DNA integrity were confirmed by genome sequencing. Xuzhou21 cured of the pO157_Sal plasmid was named as Xuzhou21m.

### Plasmid and single gene complementation of Xuzhou21m

Vector pRS551 was used as PCR template for kanamycin resistant (Km^r^) gene[56].The Km^r^ gene was inserted between pO157_Sal_36 and *nikB* gene without disrupting any open reading frame (ORF) using a one-step gene inactivation method as described by Datsenko and Wanner [57]. The km^r^ marked pO157_Sal was extracted using SDS alkaline lysis method [58] and introduced into Xuzhou21m by electroporation using gene pulse II apparatus (Bio-Rad, USA). The complemented strain was named as Xuzhou21c.

The single gene complementation (*stpA* and *hha*) strains were constructed using pBAD30 (Ap^r^) due to its lower copy number in this study. The *stpA* and *hha* genes were amplified from purified Xuzhou21 DNA template by PCR. The primers used were listed in **Table S2A**. The *stpA* and *hha* products were cloned into the *Eco*RI-*Hin*dIII site of pBAD30 and transformed by electroporation into Xuzhou21m, creating Xuzhou21m+*stpA* and Xuzhou21m+*hha* respectively. Empty pBAD30 was transferred into Xuzhou21m as negative control.

### DNA and RNA isolation

Genomic DNA were extracted from both Xuzhou21 and Xuzhou21m using Wizard Genomic DNA purification kit (Promega, Madison, WI, USA) according to the manufacturer's protocols.

For total RNA isolation, the bacteria were inoculated in 5 ml LB broth at 37°C with shaking for 16 h. 50 µl of the above culture was inoculated in 5 ml fresh LB broth and the culture was shaken at 37°C for about 2.5 h until the OD_600_ reached 0.6. 500 µl of the culture were mixed with 1 ml RNA protect bacterial reagent (Qiagen, Hilden, Germany) to stabilize RNA according to the manufacturer's instructions and was centrifuged at 5000×g for 10 minutes to pellet the cells, which were then resuspended in 100 µl TE buffer (30 mMTris·HCl,1 mM EDTA, pH 8.0) containing 15 mg/ml lysozyme and 1 mg/ml proteinase K. Total RNA were then isolated according to the standard protocol using an RNeasy mini kit (Qiagen). The RNA quantity and integrity were analyzed using Agilent 2100 Bioanalyzer (Agilent Technologies). RNA was stored at −80°C for the use of transcriptome analysis.

We extracted RNA from 3 separate biological experiments and these RNA samples were pooled for RNA-seq. We did not perform RNA-Seq for each biological replicate separately partly due to the cost of RNAseq at that time. Further Illumina RNA-seq data are highly replicable with relatively low technical variation [18].

### Plasmid copy number and major virulence genes transcriptional level

The copy number of pO157 was determined using real-time PCR with the Rotor-gene Q machine (Qiagen). The primer sequences and annealing temperatures are listed in **Table S2B**. Expression level of major virulence genes was measured by reverse-transcription quantitative PCR (RT-qPCR) using One Step SYBR PrimeScript RT-PCR Kit II (Takara, Dalian, China) in the Rotor-Gene Q machine (Qiagen). Primer sequences and annealing temperatures are listed in **Table S2C**. The relative expression level of target genes and major virulence genes, genes copy numbers were calculated as 2^−ΔΔCT^ [19]. The mRNA expression level of each target gene was normalized to the expression level of *gapA*. Each assay was performed in triplicate.

### Genome sequencing, RNA sequencing and mapping

The Xuzhou21m genome was sequenced using Illumina paired end sequencing. All reads were mapped to the complete genome in previous study (Genbank accession no. CP001925, CP001926 and CP001927) by SOAP pipeline [59].

The total RNA extracted from Xuzhou21 and Xuzhou21m were first treated with Ribo-Zero™ rRNA Removal kit to remove rRNA. The mRNA was fragmented and produced cDNA libraries primed with random hexamers. cDNA was selected by size, amplificated using PCR and then sent to sequencing using Illumina Hiseq™ 2000 commercially.

The RNA-Seq data have been submitted to GEO database and the GEO accession number is GSE44846 (http://www.ncbi.nlm.nih.gov/geo/query/acc.cgi?acc=GSE44846).

### Bioinformatics analysis and transcriptome analysis

Images generated by sequencers were converted by base calling into nucleotide sequences, which are called raw data or raw reads and are stored in FASTQ format. Reads were discarded if containing only adaptors, unknown bases more than 10% of a read, or more than half the bases of a read with quality less than 5. Reads after filtering were called clean reads, on which all following analyses were based.

Xuzhou21 genome sequence was used as the reference for RNA sequencing mapping and functional analysis. Clean reads were mapped to reference genome and genes sequences respectively using SOAP2 [60]. Mismatches no more than 2 were allowed in the alignment. The distribution of reads was plotted by its location in the reference genome, and then divided into gene region and intergenic region. Genome and gene coverage was calculated by counting the number of reads mapped to the genome and individual genes respectively. The gene expression was calculated using the RPKM method [20]. RPKM ratio and p value [61] were used to evaluate the difference between two samples. Differential expression genes (DEGs) were chosen with a P<0.05 and a ratio of 2 or greater.

### Validation of the transcriptome results by RT-qPCR

To confirm the results of the gene expression data from RNA-Seq, the expression levels of 167 selected genes that maintain higher, moderate and lower expression levels in RNA-Seq were measured using RT-qPCR. Primer sequences and annealing temperatures for these genes examined are in **Table S2D**. The *gapA* gene was used for within sample normalization.

### Statistical analysis

The results were analyzed using the statistical software package SPSS 15.0 for Windows (IBM SPSS). Statistical analysis was performed using the *t* test. Values of *P* ≤0.05 were considered statistically significant.

## Supporting Information

Table S1 **Down-/up-regulated genes in Xuzhou21.**
(XLS)

Table S2 **Primers.**
(XLS)

Table S3 **Expression level of major virulence genes in Xuzhou21 and Xuzhou21m.**
(XLS)
